# Nipah Virus Infection Generates Ordered Structures in Cellulo

**DOI:** 10.3390/v14071523

**Published:** 2022-07-12

**Authors:** Cecilia Alejandra Vázquez, Lina Widerspick, Roland Thuenauer, Carola Schneider, Rudolph Reimer, Pedro Neira, Catherine Olal, Michelle Heung, Linda Niemetz, Philip Lawrence, Indre Kucinskaite-Kodze, Lars Redecke, Beatriz Escudero-Pérez

**Affiliations:** 1 Instituto de Química Biológica de la Facultad de Ciencias Exactas y Naturales (IQUIBICEN), Consejo Nacional de Investigaciones Científicas y Técnicas-Universidad de Buenos Aires, Ciudad Universitaria, 1428 Buenos Aires, Argentina; cvazquez@qb.fcen.uba.ar; 2 WHO Collaborating Centre for Arbovirus and Haemorrhagic Fever Reference and Research, Bernhard Nocht Institute for Tropical Medicine, 20359 Hamburg, Germany; lina.widerspick@bnitm.de (L.W.); pedro.neira@bnitm.de (P.N.); olal@bnitm.de (C.O.); michelle.heung@bnitm.de (M.H.); linda.niemetz@bnitm.de (L.N.); 3 German Center for Infection Research (DZIF), Partner Site Hamburg-Luebeck-Borstel-Reims, 38124 Braunschweig, Germany; 4 Technology Platform Microscopy and Image Analysis, Leibniz Institute of Virology (LIV), 20251 Hamburg, Germany; roland.thuenauer@leibniz-liv.de (R.T.); carola.schneider@leibniz-liv.de (C.S.); rudolph.reimer@leibniz-liv.de (R.R.); 5 Advanced Light and Fluorescence Microscopy Facility, Centre for Structural Systems Biology (CSSB), 22607 Hamburg, Germany; 6 Science and Humanities Confluence Research Center (EA 1598), Catholic University of Lyon (UCLy), 69002 Lyon, France; plawrence@univ-catholyon.fr; 7 Life Science Center, Vilnius University, Sauletekio 7, LT-10257 Vilnius, Lithuania; indre.kodze@bti.vu.lt; 8 Institute of Biochemistry, Center for Structural and Cell Biology in Medicine, University of Lübeck, Ratzeburger Allee 160, 23562 Lübeck, Germany; redecke@biochem.uni-luebeck.de; 9 Deutsches Elektronen Synchrotron (DESY), Notkestrasse 85, 22607 Hamburg, Germany

**Keywords:** Nipah virus, ordered structures, in cellulo, SIM, TEM, SEM, CLEM

## Abstract

Nipah virus (NiV) is a zoonotic paramyxovirus with a fatality rate of up to 92% in humans. While several pathogenic mechanisms used by NiV to counteract host immune defense responses have been described, all of the processes that take place in cells during infection are not fully characterized. Here, we describe the formation of ordered intracellular structures during NiV infection. We observed that these structures are formed specifically during NiV infection, but not with other viruses from the same *Mononegavirales* order (namely Ebola virus) or from other orders such as *Bunyavirales* (Junín virus). We also determined the kinetics of the appearance of these structures and their cellular localization at the cellular periphery. Finally, we confirmed the presence of these NiV-specific ordered structures using structured illumination microscopy (SIM), as well as their localization by transmission electron microscopy (TEM), scanning electron microscopy (SEM), and correlative light and electron microscopy (CLEM). Herein, we describe a cytopathogenic mechanism that provides a new insight into NiV biology. These newly described ordered structures could provide a target for novel antiviral approaches.

## 1. Introduction

Nipah virus (NiV) is a zoonotic emergent virus of the *Paramyxoviridae* family, with a fatality rate reaching 92% in humans. This virus represents a threat for public health and can cause considerable economic repercussions, since it can also infect pigs [1]. Among NiV cytopathogenic mechanisms, its ability to rapidly form syncytia is a hallmark of infection. As with other members of the *Mononegavirales*, the accumulation of inclusion bodies (IB) in the cytoplasm of NiV-infected cells is a very common phenomenon [2,3,4,5]. Such IB are generally described as an aggregation of viral proteins that in some cases, can function as viral factories inside the cell [6].

Interestingly, the accumulation of certain proteins with a specific amino acid composition and disposition facilitates nucleation, thereby, in some cases, inducing their precipitation as crystals. In fact, it has been observed that such protein-derived aggregates can naturally appear in living cells; for instance, insulin crystals have been shown to form in secretory granules [7]. This process is known as in cellulo, or in vivo, crystallization and can be classified as a native or disease associated intracellular process, which can be harmless, harmful, or useful for the cell [8,9]. As a primary function, native in cellulo aggregation supports protein storage (e.g., as a nutrient supply), and encapsulation has also been described as a primary function of some in cellulo crystals [10,11]. Other functions associated with the in cellulo formation of ordered aggregates or crystal-like structures include compartmentalization [12], wound sealing [13], and the removal of toxins. However, disease-associated crystallization is believed to be a defense mechanism in response to stress or cellular damage [14,15].

While highly ordered structures may be easily observed with basic optical microscopy, super-resolution microscopy techniques, electron microscopy, or diffraction by X-ray crystallography are commonly used to define their crystalline state by visualization of the crystal lattice or detection of crystal-specific Bragg diffraction [16,17]. 

Ordered intracellular structures or crystals can assume many different shapes (e.g., rhombohedral, rhomb-dodecahedral, bipyramidal, hexagonal, or rectangular) and occur with no apparent preference for a specific cellular compartment, being frequently present in the cellular cytosol but also in the endoplasmic reticulum (ER), peroxisomes, lysosomes, nucleus, and mitochondria, as summarized by Mudogo et al. [18]. However, individual crystal-like aggregates of a specific protein usually have a growth preference for a specific compartment [19].

While in recent years, an increasing number of publications have reported in cellulo crystallization/ordered aggregation, the specific physicochemical parameters and molecular mechanisms required to generate such structures in cells have not yet been fully elucidated, and in many cases, the formation of these structures remains unperceived. In this study, we describe the presence of intracellular, rhombohedron-shaped ordered clusters during NiV infection in Vero E6 and Huh-7.5 cells. Although a typical crystal lattice is not observed internally by electron microscopy, these structures present external characteristic sharp edges, which is an indication of ordered structure. Furthermore, we provide a description of their appearance during infection, their formation kinetics, and their subcellular localization.

## 2. Materials and Methods

### 2.1. Cells and Infections

Vero E6 (ATCC, Vero C1008, clone E6, Manassas, VA, USA) and Huh-7.5 (Lonza, Cologne, Germany) cells were cultivated in Dulbecco’s modified Eagle’s medium (DMEM, PanBiotech, Aidenbach, Germany) supplemented with 100 U/mL of Pen/Strep (Gibco, Thermo Fisher Scientific, Paisley, UK) and 5% or 10% of fetal bovine serum (FBS, Gibco, Thermo Fisher Scientific), respectively.

All experiments were performed with NiV (Malaysia strain, accession number MK673562.1), EBOV (Mayinga strain, accession number KR063671.1), and JUNV (Junín XJ strain, accession number JF799981.1 and JF799977.1), under BSL-4 conditions, in the Bernhard Nocht Institute for Tropical Medicine (Hamburg, Germany). All infections were conducted at an MOI of 0.1. Briefly, 5 × 10^4^ Vero E6 or Huh-7.5 were seeded on glass coverslips in 24-well plates, and the following day, were infected for one hour at 37 °C, before removal of the inoculum and addition of DMEM supplemented with 2.5% of FCS. After the specified infection times, cells were fixed with either 4.5% formaldehyde solution (SAV Liquid Production GmbH, Flintsbach am Inn, Germany) or a 1:1 dilution of acetone:methanol (Ac:MeOH) (Sigma-Aldrich, Burlington, MA, USA and Carl Roth, Karlsruhe, Germany respectively) for one hour at room temperature (RT).

### 2.2. Immunofluorescence Analysis

A total of 5 × 10^4^ Vero E6 and Huh-7.5 were seeded onto glass coverslips (number 1, 12 mm, assistant, Sondheim vor der Rhön, Germany) in 24-well plates and infected at 70% confluency with the respective viruses. At various time points post-infection (p.i.), namely 8 h p.i., 18 h p.i., 24 h p.i., or 48 h p.i., infected cells were inactivated with either 4.5% formaldehyde solution or a 1:1 dilution of Ac:MeOH for 1 h at RT. Paraformaldehyde (PFA) inactivated samples were washed with PBS (PanBiotech, Aidenbach, Germany) 3 times for 5 min, prior to being permeabilized in 0.1% (*v*/*v*) Triton-X-100 (Carl Roth) in PBS for 10 min at RT. The Ac:MeOH inactivated samples did not require such permeabilization and were air-dried in a chemical hood. After additional washing steps, as described above, 1% (*w*/*v*) bovine serum albumin (BSA, Sigma Aldrich, Darmstadt, Germany) in PBS was added to the cells to block unspecific binding for 30 min at RT. After washing, cells were stained with the respective primary antibodies, diluted in PBS for 1 h at RT in a wet chamber. The primary antibodies used were NiV N antibody (1:750, mouse, kindly provided by Indre Kucinskaite-Kodze from the Life Science Center at Vilnius University, Vilnius, Lithuania), JUNV NP antibody (1:300 mouse, BEI Resources, Clone IC06-BE10), in-house mouse polyclonal anti–pan-Ebolavirus NP primary antibody (1:2000, 1 h at RT), anti-human Golgi Glg1 (1:50, rabbit IgG, Invitrogen—Thermo Fisher Scientific, Waltham, MA, USA, PA5-26838), and anti-human peroxisome Pmp70 (1:100, rabbit IgG, Invitrogen, PA1-650). Following extensive washing, secondary antibodies goat anti mouse FITC (1:100, Thermo Fisher Scientific, 62-6511), goat anti mouse AF647 (1:1000, Thermo Fisher Scientific, A32728), or donkey anti rabbit AF647 (Thermo Fisher Scientific, A-31573), diluted in PBS, were incubated for 1 h at room temperature in a dark, wet chamber. If applicable, actin (Phalloidin, 1X, Sigma, P5282) or microtubule (Flutax-2, 1 µM, Thermo Fisher Scientific, P22310) staining was performed during this incubation period. Upon completion of the final washing steps, cover slips were dipped into ddH_2_O, dried, and mounted onto microscopy slides using Glycerol Mounting Medium with DAPI and DABCO^TM^ (Abcam, Cambridge, UK) for counterstaining of the nuclei. Imaging was performed with Zeiss Axio Imager M1 microscopes, or a Leica SP8 confocal microscope. For image analysis, FIJI with Bio-Formats plug-in for Mac OS X was employed [20]. 

### 2.3. Quantification of NiV Ordered Structures Size 

Sizes of the organized structures observed for NiV were estimated by using standard tools and plugins provided in ImageJ (v.1.51) software. Briefly, the Straight Line tool in FIJI was used to define the length of one side of NiV ordered structures and subsequently, these lines were added as regions of interest (ROIs) to the ROI Manager. Once one side of each ordered structure was traced, its length was obtained by using the command “measure” and choosing “shape descriptors” as output. The length of one side per ordered structure was determined in 9 fields for Ac:MeOH fixation and PFA fixated samples, respectively, in three independent experiments. Size distribution was then analyzed with GraphPad Prism version 8.0.1 for Windows (San Diego, CA, USA).

### 2.4. Super-Resolution Microscopy, Structural Illumination Microscopy 

For super-resolution microscopy (SRM) via structural illumination microscopy (SIM), Huh-7.5 cells grown on cover slips (#1.5, 10 mm, Carl Roth, Karlsruhe, Germany) were infected at an MOI of 0.1 and at 18 h p.i., cells were fixed with 4.5% formaldehyde, as described above. The cells were stained according to the protocol detailed above, using NiV N primary antibody (1:500 dilution), followed by a secondary goat anti mouse antibody conjugated to FITC (1:100, Thermo Fisher Scientific, 62-6511). Fluorescence Mounting Medium (Dako, Agilent Technologies, Santa Clara, CA, USA) was used to mount the cover slips onto microscopy slides. Imaging was performed with a Nikon-N-SIM microscope with a 488 nm laser and an apochromatic 100× oil immersion objective (NA 1.49).

### 2.5. Transmission Electron Microscopy (TEM)

A total of 5 × 10^5^, 7.5 × 10^5^ or 1.25 × 10^6^ Vero E6 cells were plated in a high Grid-500 35 mm μ-Dish (IBIDI). Cells were infected at an MOI of 0.1, and after 24 h, cells were fixed for 1 h in 4.5% PFA and postfixed with 2.5% glutaraldehyde (GA) overnight. Subsequently, cells were washed with PBS, postfixed for 30 min with 1% OsO_4_ in PBS, washed with ddH_2_O, and stained with 1% uranyl acetate in water. The samples were gradually dehydrated with ethanol and embedded in Epon resin (Carl Roth, Karlsruhe, Germany) for sectioning. Ultrathin 50 nm sections were prepared using an Ultracut Microtome (Leica Microsystems, Weitzlar, Germany). The sections were poststained with 1% uranyl acetate. Electron micrographs were obtained with a 2 K wide angle CCD camera (Veleta, Olympus Soft Imaging Solutions GmbH, Münster, Germany) attached to a FEI Tecnai G 20 Twin transmission electron microscope (FEI, Eindhoven, The Netherlands) at 80 kV.

### 2.6. High Resolution Scanning Electron Microscopy (SEM)

After 24 h p.i., NiV-infected Vero E6 cells were fixed with 4.5% formaldehyde and were permeabilized in 0.1% Triton-X-100 in PBS for 10 min at RT, stained according to the protocol detailed above, using NiV N primary antibody (1:500 dilution), followed by a secondary goat anti-mouse antibody conjugated to FITC. Samples were processed and analyzed for TEM, as described above. For SEM, samples were dehydrated in a graded series of ethanol, air-dried after transition to pentane, and finally sputtered with 5 nm gold. Images were obtained in a Tescan Clara scanning microscope operated at 3 kV, using an ET secondary electron detector.

### 2.7. Correlative-Light Electron Microscopy (CLEM)

A total of 5 × 10^4^ Vero E6 cells were seeded in a high Grid-500 35 mm μ-Dish (IBIDI) and were infected with NiV at an MOI of 0.1 24 h later. At 24 h post-infection, the dishes containing NiV infected cells were fixed for 1 h in 4.5% formaldehyde. The inactivated samples were washed with PBS 3 times for 5 min, prior to being permeabilized in 0.1% (*v*/*v*) Triton-X-100 in PBS for 10 min at RT. Then, cells were blocked with 1% BSA in PBS for 30 min at RT. After washing, cells were stained with NiV N antibody for 1 h at room temperature in a wet chamber. Following extensive washing, secondary antibodies goat anti mouse FITC (1:100) were incubated for 1 h at room temperature in a dark, wet chamber. Fluorescence imaging was carried out with a spinning disc microscope (Nikon Ti2E equipped with a Yokogawa CSU-W1 spinning disc unit, an Andor iXon Ultra EMCCD camera, and a 100× NA 1.49 plan apo oil immersion objective. The samples were gradually dehydrated with ethanol and embedded in Epon resin (Carl Roth, Germany) for sectioning. Imaging was performed using a FEI Tecnai G 20 Twin transmission electron microscope. The samples were also processed for TEM, as described above. Electron micrographs were obtained with a 2K wide angle CCD camera (Veleta, Olympus Soft Imaging Solutions GmbH, Münster, Germany) attached to a FEI Tecnai G 20 Twin transmission electron microscope (FEI, Eindhoven, The Netherlands) at 80 kV.

### 2.8. Protein In Silico Analysis

The amino acid sequences of NiV (AAX51852.1), EBOV (AAD14590.1), and JUNV (ACS12871.1) nucleoproteins and NiV matrix protein (NP_112025.1) were analyzed with ProtParam (https://web.expasy.org/protparam/, accessed on 8 December 2021) software from Expasy to determine the percentage of hydrophobic amino acids contained in their sequences [21]. In order to estimate their solubility index, Protein-Sol (https://protein-sol.manchester.ac.uk, accessed on 8 December 2021) software from Expasy was used [22].

The CamSol method from The Chemistry of Health Software (https://www-cohsoftware.ch.cam.ac.uk/index.php/camsolintrinsic, accessed on 29 December 2021) was used for the calculation of the intrinsic solubility profile and the score of each analyzed protein [22]. Monomeric 3D model structures of NiV N, NiV P, and NiV M were calculated with Alphafold software (https://alphafold.ebi.ac.uk, accessed on 8 December 2021, [23,24]) using the associated protein sequences with the accession numbers AY858110.1, HM545087.1, and NP_112025.1, respectively. The Swiss-Model server (https://swissmodel.expasy.org/interactive, accessed on 8 December 2021) was used for modeling of the 3D structures of the multimeric forms of NiV N, NiV P, and NiV M. Among the 8 templates available for NiV N in the Protein Data Bank (PDB; www.rcsb.org, accessed on 8 December 2021), the selected template, PDB 7NT5 obtained by cryoEM at 3.5 Å resolution, showed an identity of 98.68%, coverage of 1.00, QMEAN: 0.81 ± 0.05, global model quality estimation (GMQE) of 0.76, and quaternary structure quality estimation (QSQE) of 0.78. For structural modeling of NiV P, 28 templates are available in the PDB. The selected template 4GJW was obtained by X-ray crystallography at 3.0 Å resolution; it showed an identity of 99.08%, coverage of 0.15, QMEAN: 0.72 ± 0.05, GMQE: 0.06, and QSQE: 0.40. Among the 27 templates available for NiV M, the selected sample 6BK6 was obtained with X-ray crystallography at 2.5Å resolution, showing an identity of 91.81%, coverage of 0.97, QMEAN: 0.86 ± 0.05, GMQE: 0.84, and QSQE: 0.82. Pymol software was used to generate 3D protein structure images and videos [25].

## 3. Results

### 3.1. Formation of in Cellulo Ordered Structures during NiV Infection

**Figure 1:**
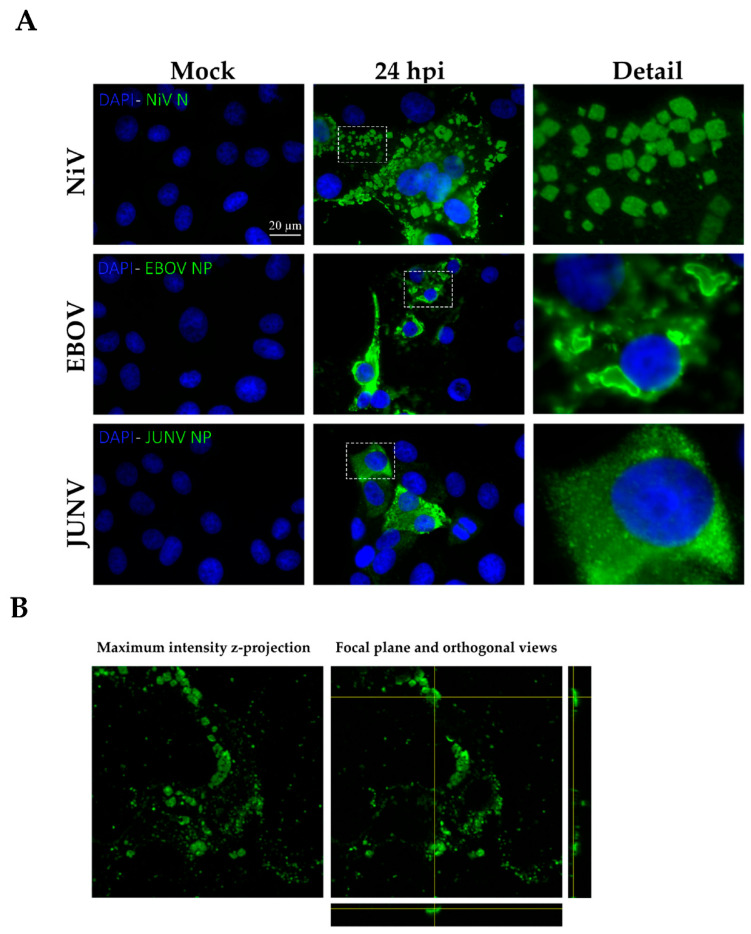
**The ordered structure formation is virus-specific.** (**A**) Microphotographs of Vero E6 cells infected with NiV, EBOV or JUNV. Viral nucleoproteins are immunolabeled in green, while nuclei are shown in blue; scale bar: 20 µm. In the right panel, magnifications of intracellular distribution of the nucleoproteins in infected cells correspond to the areas marked in white dotted boxes in the middle column. Three independent experiments were performed, in triplicate. (**B**) A z-stack of NiV infected cells, with NiV ordered structure formations, was generated. In the left panel: maximum intensity projection of the z-stack, where the maximum value of each focal plane is plotted. In the right panel: one focal plane of the z-stack and orthogonal views of the z-stack along the planes, shown in yellow, where a cross-section of a single ordered structure is depicted.

While RNA viruses share plenty of features during their cellular infection cycles, the exact processes may differ in terms of replication, cellular transmission, and immune evasion mechanisms that take place during infection. The accumulation of viral proteins in cells, normally as inclusion bodies, is a frequent occurrence during infection with *Mononegavirales* viruses [2,3,4,5]. To determine whether the formation of aggregated structures in cellulo is a process specific to one or several viruses, we infected Vero E6 cells with two members of the *Mononegavirales* order*,* Ebola virus (EBOV) and Nipah virus (NiV), and one member of the *Bunyavirales*, Junín virus (JUNV). As shown in Figure 1A, only NiV infection yielded a highly inhomogeneous distribution of the viral nucleoprotein N in the cytoplasm of the infected cells after 24 h of infection with immunofluorescence. A more detailed magnification revealed the formation of specific, apparently ordered, structures characterized by sharp edges, which is usually characteristic of crystalline structures. Thus, these aggregates are denoted as ordered, or “rhombohedral-like,” structures hereafter. EBOV and JUNV infected cells did not present any indications for the formation of similar ordered structures (Figure 1A).

In order to determine the three-dimensional shape of the observed NiV structures, confocal microscopy experiments were performed to allow for the reconstruction of cells and the ordered structures. We acquired z-stacks, with a distance of 0.38 µm between focal planes, and used FIJI for the reconstruction. The orthogonal views crossing through a single NiV ordered structure show that they are generally flat and do not extend into the z-direction (Figure 1B).

### 3.2. Comparison of Fixation Methods for NiV in Cellulo Ordered Structure Detection

**Figure 2:**
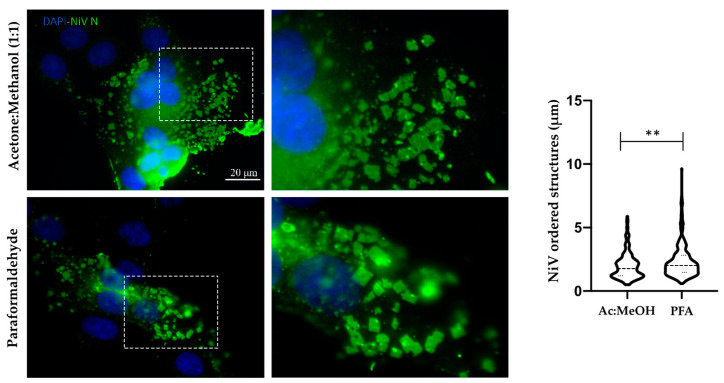
**Sample fixation method does not affect NiV rhombohedron structure formation.** On the left, microphotographs of NiV infected Vero E6 cells fixed with acetone:methanol (Ac:MeOH) or 4.5% paraformaldehyde (PFA). NiV nucleoprotein (N) is labeled in green and nuclei in blue; scale bar: 20 µm. Panels in the middle column represent enlarged versions of the inset box. On the right, a representative quantification of ordered structure size in samples fixed with either Ac:MeOH or 4.5% PFA is depicted. A total of 9 images from 3 independent experiments per method were used. A non-parametric Mann–Whitney test showed significant differences between the fixation methods. Significance is indicated by ** for *p* < 0.01.

Treatment of samples with fixation and permeabilization reagents can, on occasion, generate artifacts during cellular preservation processes. In order to exclude the possibility that the ordered structures observed during NiV infections were generated due to the fixation agent used, two different chemical fixation reagents were tried: Ac:MeOH and 4.5% PFA. While the former is a precipitating fixative, aldehydes are additive fixatives, and thus, they have very different physiochemical properties [26]. We were able to detect intracellular ordered structures after fixing NiV infected cells with either reagent, thus ascertaining that these structures were not artifacts of the fixation process (Figure 2, left and middle). Interestingly, the rhombohedron-shaped structures show regular edges and a very wide size distribution, ranging from less than 1 μm up to more than 10 μm. Despite the broad distribution range, the majority of NiV rhombohedral-like structures are characterized by an edge length between 1–3 μm. Although the shape of the ordered structures in NiV-infected cells did not seem to vary between the fixation methods, the size of the ordered structures is, on average, slightly increased in PFA fixed samples (Figure 2, right). However, while statistically significative, the biological relevance of this difference is negligible, given that the mean length of these structures was 1.98 µm ± 1.04 in samples fixed with Ac:MeOH and 2.34 µm ± 1.29 in samples fixed with 4.5% PFA. Moreover, the quartiles (Q) of the ordered structures sizes were Q1 (first quartile) = 1.20 µm and Q3 (third quartile) = 2.40 µm in Ac:MeOH-treated samples, and Q1 = 1.47 µm and Q3 = 2.81 µm in PFA-treated samples. 

### 3.3. Kinetics of NiV Ordered Structures Formation and Location in the Cell

**Figure 3:**
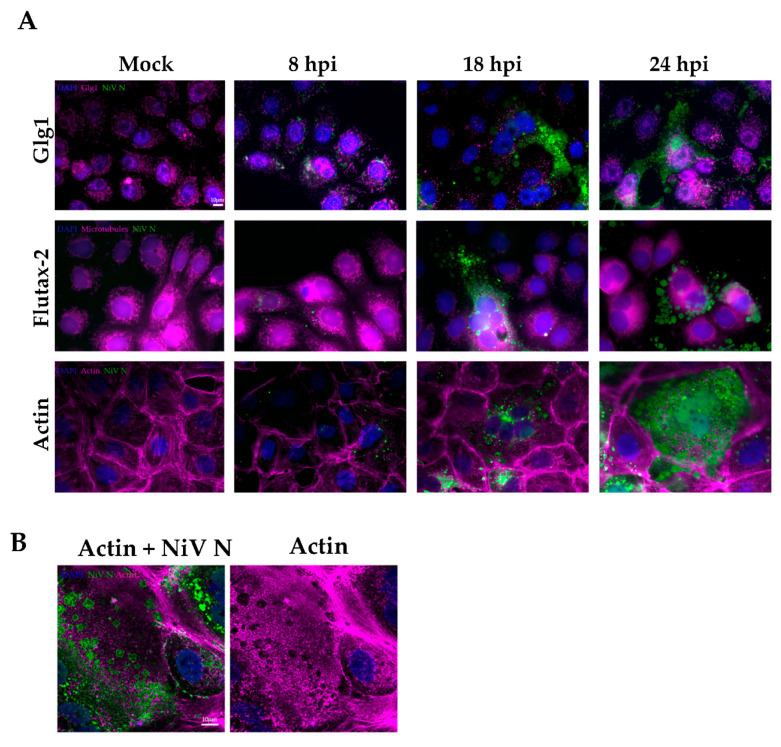

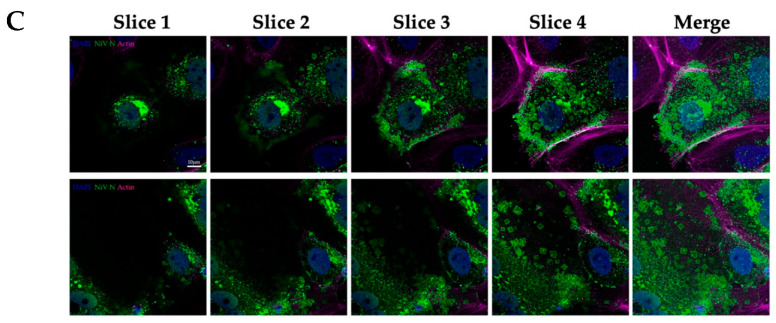
**Kinetics of the formation of ordered structures and lack of colocalization of NiV N in different cellular structures.** (**A**) Vero E6 cells were fixed at 8, 18, and 24 h post infection (hpi), and relative localization of Golgi (Glg1, magenta), microtubules (Flutax-2, magenta) and actin (Phalloidin, magenta), together with NiV N (green) are shown. Cellular nuclei are stained with DAPI (blue); scale bar: 10 µm. (**B**) Vero E6 staining with NiV N (green) and actin (Phalloidin, magenta); scale bar: 10 µm. (**C**) Z-slices of a z-stack from Vero E6 cells stained with NiV N (green) and actin (Phalloidin, magenta); scale bar: 10 µm.

In cellulo aggregation can take place at different growth rates in organelles or in the cellular cytoplasm. In order to determine when and where the formation of NiV ordered structures occurs, a kinetics analysis of the infection process was performed, and the Golgi, microtubules, and actin were co-stained together with NiV N (Figure 3A). Vero E6 cells infected with NiV were fixed at 8, 18, and 24 h p.i., and NiV ordered structures were evidenced. While in some instances, such ordered clusters with well-defined edges were observed as early as 8 h p.i. (Figure S1), this was rather rare, and their appearance increased with time. The highest number of the organized, rhombohedral-like NiV structures were detected 24 h p.i. At 48 h, the cell monolayer was too damaged, with hardly any cells left for imaging. 

Concerning their localization, it would appear that the ordered structures do not colocalize with the cellular structures tested, namely the Golgi, actin, and microtubules. In addition, there was no evidence of colocalization between NiV N and the peroxisomes (Pmp 70 marker, Figure S2). Since NiV N does not colocalize with any other cellular structure, we assumed that NiV N ordered structures accumulate in the cellular cytoplasm. Moreover, the absence of apparent colocalization with microtubules (Flutax-2 marker) or actin (actin marker) would indicate that these cellular polymers are not acting as a framework for structure formation (Figure 3A). In fact, it appears that NiV N aggregates impact the actin cytoskeleton distribution by physically propelling the filaments to areas surrounding the rhombohedron structures (Figure 3B).

Serial focal planes of a z-stack of NiV infected cells were taken from confocal images at 18 h p.i. These images would appear to show that the NiV N protein accumulates in the cytoplasm surrounding the nucleus. However, it is mostly in the syncytia periphery that the rhombohedral structures aggregate (Figure 3C). 

### 3.4. Analysis of NiV Infected Cells with SIM, TEM, and SEM

**Figure 4:**
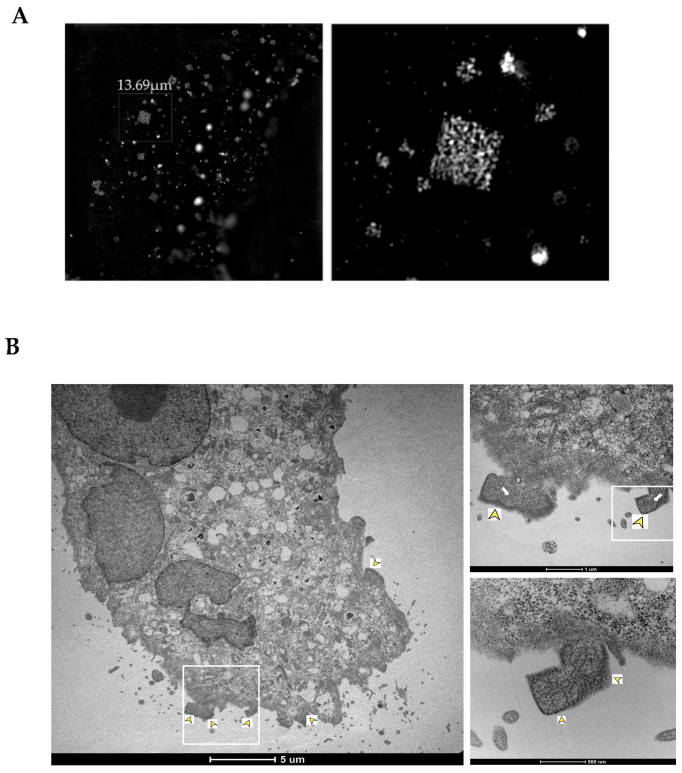

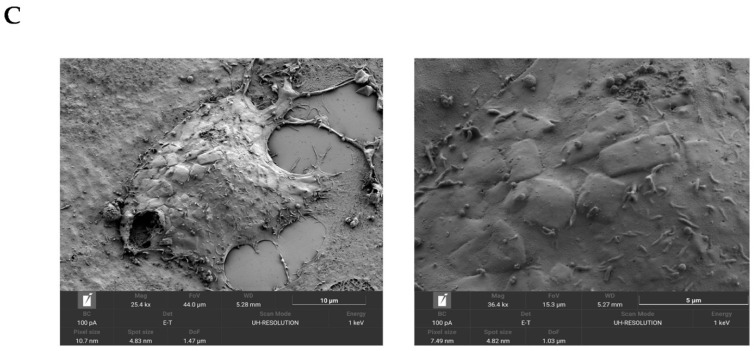
**Detection of NiV N ordered structures with SIM, TEM, and SEM.** (**A**) SIM was used to determine the regularity of single-stained entities within the NiV ordered structures, with super-resolution light microscopy. The images on the right show the magnification of the areas outlined by a rectangle in the images on the left. (**B**) TEM-overview of a NiV-infected Vero E6 cell, with magnified areas showing the ordered structures (yellow arrow heads) and RNCs (white thick arrows). (**C**) Scanning electron microscopy (SEM) of NiV-infected Vero E6 cells. NiV organized structures are displayed on the surface of the cell. In the right image, a magnification of the area in the left image showing the NiV rhombohedron-like structures.

Structured illumination microscopy (SIM) can achieve nearly 100 nm resolution inside living cells. Using SIM, we were able to determine that NiV N appears to accumulate in semi-regular rhombohedron patterns (Figure 4A). These structures exhibited straight borders and sharp edges. As expected for ordered structures, the NiV rhombohedrons also presented a semi-regular pattern. Thus, super-resolution microscopy using SIM supported our previous observations of the formation of ordered structures linked to an accumulation of NiV N.

When NiV infected Vero E6 cells were analyzed by transmission electron microscopy (TEM), which can achieve a higher resolution than SIM, ordered rhombohedral structures, similar to the ones described before, were again observable close to the membrane of infected cells (Figure 4B, yellow arrow heads). These structures, which were identified in several cells, as well as multiple times in the same cell, appear to contain multiple ribonucleocapsid (RNC) particles (thick white arrows) that can reach a size of up to 1 μm. Productive infection was confirmed (Supplementary Figure S3) by the observation of NiV nucleocapsid cross-sections (thin black arrows), as well as of virions budding from the cell surface, or pleomorphic particles released from the cells (white arrow heads). However, a highly ordered, regular lattice could not be detected by our TEM analysis. The visualization of such a lattice is a valid confirmation of the presence of protein crystals in living cells, as previously shown [27]. In this sense, both SIM and TEM identified organized rhombohedral-shaped structures in the peripheral membrane of NiV-infected cells that are not classified as regular crystals.

When NiV-infected cells were analyzed by scanning electron microscopy (SEM), the cells presented clearly visible, rhombohedral protrusions on the surface, corresponding to the ordered clusters described above (Figure 4C and Supplementary Figure S4). These structures were shown to be numerous and to form aggregates in peripheric cellular regions where their appearance tends to concentrate. Measurements of the ordered structures showed a thickness of approximately or less than 0.2 µm and an average size of more than 2 μm (in some cases, up to 4 μm), thus being larger than the virions released from infected cells, which diminishes the possibility that these structures correspond to inclusion bodies, as suggested in previous studies [28].

### 3.5. Analysis of NiV-Infected Cells with CLEM

**Figure 5:**
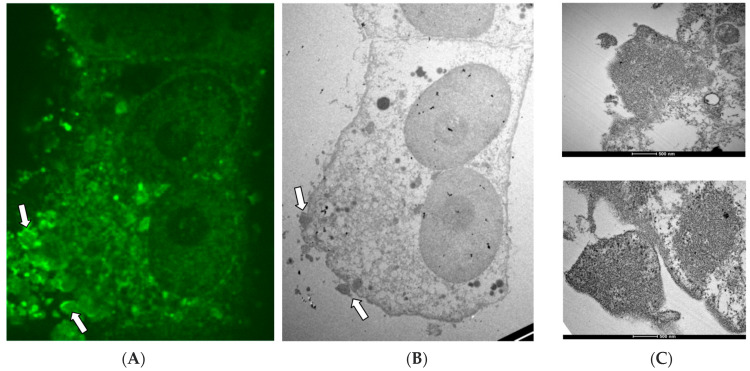
**Detection of NiV N ordered structures with CLEM.** NiV infected Vero cells were analyzed by fluorescence (**A**) and electron microscopy images (**B**,**C**) simultaneously. (**A**) NiV-infected cells, where NiV N is detected by spinning disc confocal microscopy. NiV ordered structures are indicated by white arrows. (**B**) The same syncytium as in image (**A**) is analyzed by electron microscopy. White arrows indicate NiV ordered structures. (**C**) The image on the right shows magnification of the NiV ordered clusters indicated with white arrows in images (**A**,**B**).

In order to acquire simultaneously functional and structural information, the CLEM technique was used to analyze NiV-infected samples. As shown in Figure 5A, rhombohedral-like organized specific NiV N structures are again observed. These ordered clusters have well defined edges, like those commonly observed in crystal-like structures. Investigation of the same syncytia by electron microscopy (Figure 5B) confirmed the shape of the NiV clusters. A more detailed view of these organized structures (Figure 5C) revealed that the high fluorescence intensity, and thus, the high abundance of NiV N, corresponds to the most ordered fraction of the clusters, forming an organized cellular/viral ‘wall’ that surrounds, in part, the building blocks of the clusters. The detailed electron microscopy image on the right also shows that the NiV ordered structure content corresponds to the NiV RNC that do not contain a highly regular lattice structure. Importantly, it would appear that when the rhombohedral organization is disrupted, they lose their regular ordered shape and defined edges (C), and the intensity of NiV N expression is also diminished. Thus, again with these images the ordered clusters are not classified as crystalline.

### 3.6. In Silico Analysis

Although several factors might contribute to the in cellulo aggregation of proteins, their hydrophobic amino acid content plays a role in the case of protein aggregation. Therefore, we determined the percentage of hydrophobic amino acids in the nucleoprotein sequences of NiV, EBOV, and JUNV, but also in the NiV matrix M protein, combined with the predicted scaled solubility (Table S1). Unlike the NiV P protein, other studies have previously shown that the matrix NiV protein M determines the location of NiV inclusion bodies in the periphery of the cell [28], in a similar localization as the NiV ordered structures observed in this study. Thus, we hypothesized that the matrix protein could play a role in their formation and therefore, included the viral M protein in this analysis.

The percentage of hydrophobic amino acids was shown to be higher in NiV N and NiV M than in the other viral proteins, resulting in a predicted scaled solubility lower that the average of proteins. This is calculated with Protein-Sol software, where the population average for the dataset is 0.45. Thus, proteins with values greater than 0.45 are predicted to have a higher solubility (and less aggregation probability) than the average proteins, and proteins with values lower than 0.45 are predicted to be less soluble and therefore, are more prone to form crystals. Consequently, the predicted scaled solubility of NiV N and NiV M, which was found to be lower than 0.45, hints towards an increased tendency for crystallization. In contrast, JUNV and EBOV nucleoproteins show a higher predicted solubility compared to NIV N and M. The value for the EBOV nucleoprotein is even higher than 0.45, suggesting a higher solubility than the population average.

**Figure 6:**
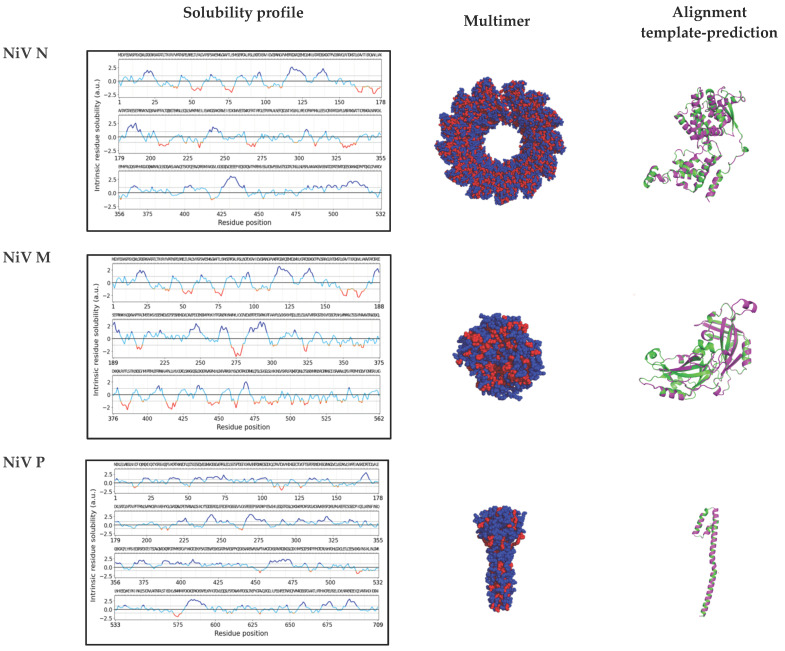
**Solubility and three-dimensional analysis of NiV N, M, and P**. On the left, solubility profiles of NiV N, NiV M, and NiV P are shown. The amino acid sequences of the NiV nucleoprotein N, the matrix protein M, and the phosphoprotein P were analyzed using the CamSol method in order to generate a solubility profile. Each amino acid in the protein sequence was assigned a score. Regions with scores higher than 1 denote highly soluble regions, while scores smaller than −1 indicate poor solubility. In the middle and on the right, three-dimensional (3D) representations of both: (i) the multimeric structure of NiV N, NiV M, and NiV P, with hydrophobic amino acids shown in red and non-hydrophobic amino-acids shown in blue, and (ii) the alignment of the NiV N, NiV M, and NiV P predicted monomeric structure (in magenta) and the monomeric template (in green) used in (i). The α-helices and β-sheets are indicated as ribbons and wide arrows, respectively.

The solubility profile calculated for the NiV N, M, and P sequences is shown in the left graphs of Figure 6. To provide these values, the CamSol method was applied, where the intrinsic solubility profile and a score of each amino acid (in an unfolded state) is calculated. With this method, amino acid regions with a score higher than 1 denote highly soluble regions, whereas regions with values lower than −1 (indicated with a dotted line and red curves) denote poorly soluble parts of the structure. As shown in the figure, NiV P does not exhibit many regions with values below −1 (in red), whereas NiV N and NiV M contain a higher abundance of poorly soluble regions with values below −1, again indicating an increased probability for insolubility and aggregate formation.

In addition, the surface exposure of the less soluble regions, which is a requirement for an increased aggregation tendency, was predicted by the structural modeling of NiV proteins (Figure 6, middle). The calculated 3D models show multimeric assemblies, which are assumed to be the biological structures present during infection. While NiV P tends to form tetramers [29], N protein encapsidates the newly synthesized viral RNA, thus forming a helical nucleocapsid assembly [30]. NiV M protein is known to form stable dimers that tend to associate into pseudotetrameric arrays which underly the plasma membrane [31,32]. While the modeling of NiV N and NiV M is available with a high coverage, it should be mentioned that due to the flexible character of NiV P, it is not possible to predict all the structure with certainty. Thus, the sequence coverage of this protein is low, and only the reliable part of the structure is modeled and displayed. The alignment between the monomeric NiV N, NiV M, and NiV P predicted structure (in magenta) and the respective monomeric template (in green) used for the multimeric representation is also displayed in the right section of Figure 6. Compared to NiV P, NiV N and NiV M show an increased surface exposure of hydrophobic amino acids (in red), which could contribute to the observation from a previous study that NiV N and NiV M, but not NiV P, appear to be viral ordered structures [28]. A video animation of the three-dimensional NiV N, NiV M, and NiV P structure predictions is presented in Video S1. When modeling the proteins, it appears that the exposure of the hydrophobic amino acids in NiV N and NiV M is higher when compared to the exposure in NiV P.

## 4. Discussion

Despite the intracellular formation of well-ordered assemblies, including protein crystals, being a known phenomenon for more than a century [33], this represents a relatively unknown process that frequently occurs unnoticed. The presence of ordered or crystal-like structures may be observed in living cells, but they are not recognized as such. This may be the case for NiV infected cells, in which two different “inclusion body” types have been described to date: perinuclear IB (IB_peri_) and plasma membrane IB (IB_pm_) [28]. Based on our findings, we believe that what were previously referred to as IB_pm_, are not, in fact, inclusion bodies, but distinct, rhombohedron-shaped NiV-ordered viral structures.

In order to visualize biological samples in detail through microscopy, it is necessary to fix them. The fixation process used is crucial, since it determines the level of resemblance between the image seen in the microscope and the in vivo structure. Thus, it is important to avoid the generation of artifacts due to sample manipulation. There are several ways to carry out chemical fixation, but some of the most commonly used methods for microscopy include the use of aldehydes and alcohols. Aldehydes are additive fixation solutions that generate covalent interactions (cross-links) between proteins and preserve their natural structure. In comparison with other aldehydes, such as glutaraldehyde, paraformaldehyde is known to have less effect in terms of the masking of a protein’s antigenicity during fixation; therefore, it is commonly used [26]. On the other hand, alcohols are denaturing or precipitating fixators which can denature the protein when they reduce their solubility, thus modifying their tertiary structure; alcohols are frequently used in combination with other chemicals such as acetone in order to improve the fixation process [26,34].

In our study we compared both types of chemical fixation, additive and denaturing, in order to eliminate the possibility that the formation of NiV-ordered structures is artificially associated with the fixation process. Moreover, we fixed cells infected by other viruses, one from the same *Mononegavirales* order (EBOV) and one from *Bunyavirales* (JUNV), using both fixation methods in order to determine the specificity of ordered structure formation during NiV infection. We observed that these structures specifically occur in NiV-infected cells, independent of the fixation method.

Several factors, including cellular elements, may contribute to this process during NiV infection; while it is not the only contributing intrinsic factor, the presence of hydrophobic amino acids, particularly at the surface of the viral protein structures, may facilitate the unique, ordered aggregation of the NiV N and M proteins [35,36]. This is in line with a previous study that confirmed the presence of NiV N and M in comparable ordered structures detected after NiV infection, even if they have been classified as IBs [28]. Consistent with less exposure of hydrophobic patches and the increased predicted solubility, no indications of the presence of NiV P were found in these structures.

While the intrinsic solubility of a protein is relevant to determine its aggregation tendency, other factors such as the specific cellular physicochemical environment, the content, polarity and disposition of the amino acids, the levels of gene expression, and the viral complexity itself play a major role in this process. It has been shown that intracellular aggregation first requires a high local concentration of the protein, along with precipitant agents, in order to render aggregation thermodynamically favorable, thus reaching the required protein supersaturation [18]. NiV N and M proteins are abundantly produced during NiV replication, and importantly, we have shown that an increase in NiV N over time coincides with the formation of the specific NiV N-ordered structures. However, it has been reported that the NiV matrix protein M is additionally required to form comparable ordered structures, previously described as IB_pm_ [28]. Indeed, it has been shown by others that the expression of NiV N protein alone or in combination with part of the NiV P protein does not induce the formation of characteristic structures in transfected cells [37]. Thus, it seems that both NiV N and NiV M are involved in or affected by this aggregation process.

The localization of intracellular aggregates in cells can be very variable, as they can appear either in cellular organelles or in the cytoplasm. NiV ordered structures were found to be exclusively located in the cytoplasmic compartment of the cell, particularly in the periphery of infected cells, or associated with syncytia. Whether they form there for strategic functional reasons, or are simply placed there by physiological forces remains to be determined. While it has been suggested that analogous NiV structures (described as IB_pm_) may have a role related to virion assembly and budding, it has not been proven that fusion events take place between IB_pm_ and the cellular membrane [28]. In contrast to this theory, our findings suggest that NiV-ordered structures may displace the actin cytoskeleton, which usually plays an important role during membrane fusion and viral syncytia formation [38,39]. The involvement of actin in syncytia formation and viral release has previously been shown for orthomyxoviruses (namely influenza) and other paramyxoviruses members, such as respiratory syncytial virus (RSV) [40,41]. Importantly, our observation of NiV-ordered structures of up to 10 µm in size suggests that a role related to multiplicity and the formation of inclusion bodies, as suggested in previous studies [28], is dubious. However, the fact that regular NiV clusters are observed by TEM and SEM exclusively in the periphery of the cellular membrane does not negate the possibility that these structures are related to the budding processes. Indeed, the fact that NiV-organized structures have a rhombohedral form that can be disrupted during budding provides a new insight of how these structures act during NiV infection.

This study provides a detailed physical characterization of NiV-organized structures that could help to further investigate their role, which is likely of functional importance. In addition, the aggregation of viral proteins may be a virulence factor, since this phenomenon concentrates viral proteins over time, but could also represent a defensive mechanism of the virus against proteolytic degradation. While it is suspected that such an abnormal protein assembly mechanism conveys a viral strategy which may have been evolutionary optimized, an accidental event cannot be excluded either. In fact, NiV protein aggregation may well be a defense mechanism of the cell, preventing cell cytotoxicity, or alternatively, have a more specific function related to viral infection, transmission, or replication. While it is possible that crystal formation could be advantageous for cells in some cases, the high concentration and size of NiV in cellulo-ordered structures may be detrimental to the functions of the cell. Consequently, it would be interesting to observe if such structures are formed in vivo.

The presented data confirms a specific degree of order in the NiV clusters, but does not allow for confirmation of the crystalline character of the detected structures. Indeed, we were unable to clearly visualize a well-ordered lattice structure by TEM, or to detect specific Bragg diffraction of X-rays at the P14 beamline using the synchrotron source PETRA III (German Electron Synchrotron DESY, Hamburg, Germany; data not shown), which are both accepted as a proof for crystallinity. Despite the high brilliance of the X-rays provided, the latter might be limited by the extremely small diffractive volume of the structures [16]. However, the characteristic rhombohedral shape of the distinct structures, combined with the frequently observed and the extremely sharp edges, strongly indicate a high degree of order.

Moreover, the ordered structures appear to be virus-specific and are not formed as artifacts of the fixation process, supported by their association with high aggregates of the NiV nucleoprotein and the numerous RNC-like structures seen with electron microcopy. The specific location in the cellular periphery suggests that this preferred localization might be due to physiological constraints. Further studies involving in vitro and in vivo analysis techniques are required to understand the molecular features promoting and regulating the formation of intracellular ordered structures during NiV infection. Our data highlights the frequency with which NiV-ordered structures appear during infection and emphasizes the importance of investigating their role in more detail, thus providing a potential target for novel therapeutic directions, with the aim of preventing their formation or disrupting these potentially detrimental complexes during NiV infection.

## Supplementary Materials

The following supporting information can be downloaded at: https://www.mdpi.com/article/10.3390/v14071523/s1, Figure S1: Incipient ordered structures in Huh-7.5 cells; Figure S2: Localization of NiV N and peroxisomes; Figure S3: Electron micrographs of NiV infected cells; Figure S4: Transmission electron micrographs of NiV infected cells containing ordered structures; Table S1: Amino acid analysis of NiV N, NiV M, EBOV NP, and JUNV NP; Video S1: Three-dimensional rotation view of NiV N, M, and P proteins.
