# Supplementary material for: Nipah Virus Infection Generates Ordered Structures in Cellulo
Source: Viruses. 2022 Jul 12;14(7):1523. doi: 10.3390/v14071523 (PMC9317923; doi:10.3390/v14071523)
Supplement: Supplementary file 1 [file viruses-14-01523-s001.zip › viruses-1685518-supplementary.pdf]

## Supplementary Figures

### Figure S1

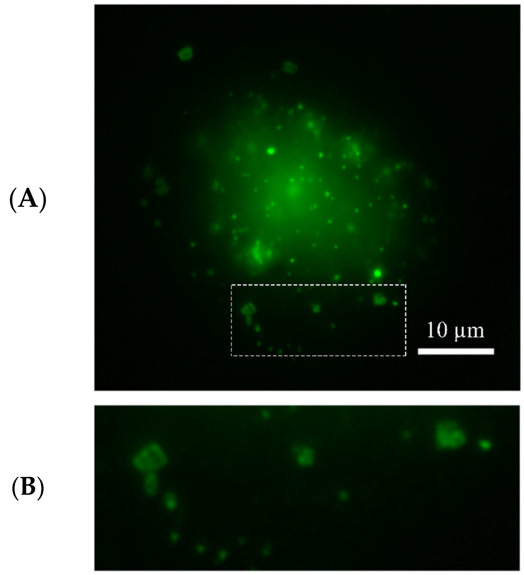


**Figure S1. Incipient ordered structures in Huh-7.5 cells.** (**A**) NiV N stain (in green) in NiV infected cells at 8h p.i. Scale bar: 10 µm (**B**) Detail of the early NiV ordered structures present in the dotted rectange

### Figure S2

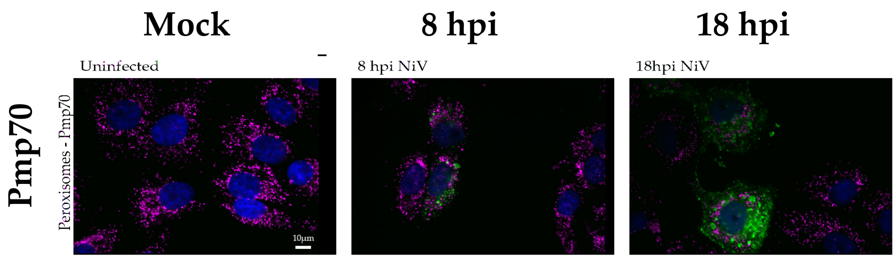


**Figure S2. Localization of NiV N and peroxisomes.** Vero E6 cells were fixed at 8-, and 18- h p.i. and localization of peroxisomes (in magenta, Pmp70) and NiV nucleoprotein N (green), were shown. Cellular nuclei were stained with DAPI (blue). Scale 10 µm.

**Figure S3**

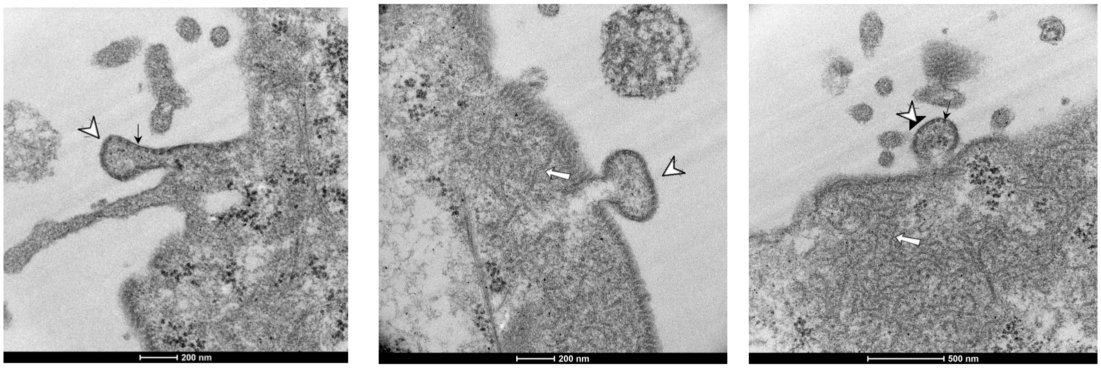


**Figure S3. Electron micrographs of NiV infected cells.** NiV particles budding (white arrow heads) and RNCs in longitudinal (white thick arrow) and cross-section (black thin arrow).

**Figure S4**

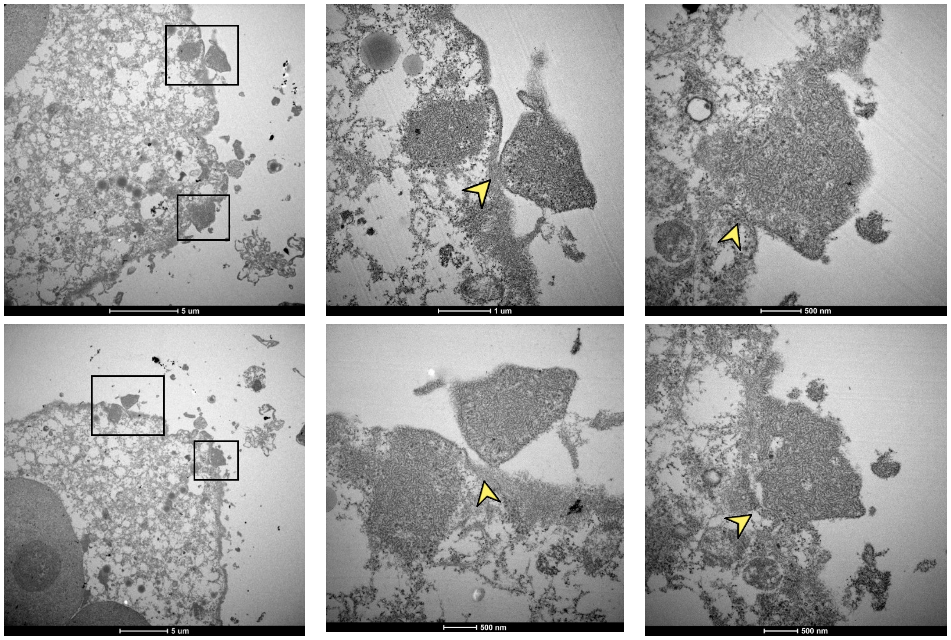


**Figure S4. Transmission electron micrographs of NiV infected cells containing ordered structures.** TEM-overview of a NiV-infected Vero E6 cell with magnified areas (in black squares) with ordered structures (yellow arrow heads).

**Table S1**

**Table 1. Amino acid analysis of NiV N, NiV M, EBOV NP and JUNV NP.**

| Amino acid (aa) | NiV N | NiV M | EBOV NP | JUNV NP |
|---|---|---|---|---|
| | Percentage | Percentage | Percentage | Percentage |
| **Ala (A) *** | 10.9 | 4.3 | 7.2 | 5.1 |
| **Arg (R)** | 7.1 | 6.0 | 4.5 | 6.0 |
| **Asn (N)** | 3.8 | 5.4 | 4.5 | 4.6 |
| **Asp (D)** | 4.9 | 5.4 | 8.0 | 6.7 |
| **Cys (C)** | 0.0 | 1.7 | 0.4 | 1.2 |
| **Gln (Q)** | 4.1 | 2.6 | 7.2 | 5.5 |
| **Glu (E)** | 7.3 | 4.8 | 8.0 | 5.5 |
| **Gly (G) *** | 7.0 | 7.4 | 5.6 | 7.3 |
| **His (H)** | 0.4 | 1.7 | 4.1 | 1.8 |
| **Ile (I) *** | 6.0 | 8.2 | 3.9 | 4.8 |
| **Leu (L) *** | 8.6 | 9.4 | 9.1 | 11.5 |
| **Lys (K)** | 4.5 | 7.7 | 5.1 | 7.4 |
| **Met (M) *** | 3.4 | 3.4 | 2.6 | 2.5 |
| **Phe (F) *** | 3.6 | 4.8 | 3.5 | 3.0 |
| **Pro (P) *** | 3.8 | 4.0 | 5.7 | 4.6 |
| **Ser (S)** | 9.8 | 8.8 | 6.5 | 7.3 |
| **Thr (T)** | 6.2 | 3.4 | 5.1 | 5.1 |
| **Trp (W) *** | 0.9 | 1.1 | 0.5 | 1.1 |
| **Tyr (Y)** | 2.1 | 4.0 | 2.8 | 2.3 |
| **Val (V)*** | 5.6 | 6.0 | 5.7 | 6.6 |
| **Pyl (O)** | 0.0 | 0.0 | 0.0 | 0.0 |
| **Sec (U)** | 0.0 | 0.0 | 0.0 | 0.0 |
| **Hydrophobic aa %** | 49.8 | 48,6 | 43,8 | 46,5 |
| **Predicted scaled solubility** | 0.349 | 0.362 | 0.544 | 0.415 |

* Hydrophobic amino acids.

**Video S1**

**NiV N**

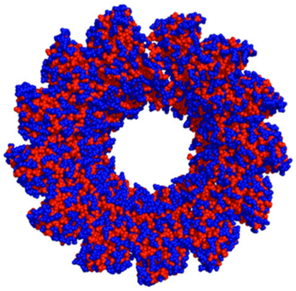

**NiV M**

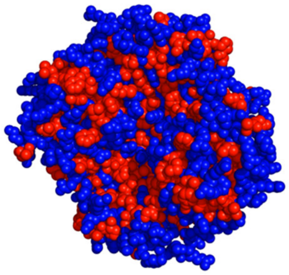

**NiV P**

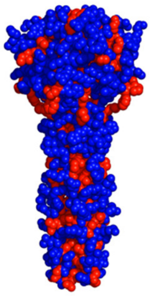

**Video S1. Three-dimensional rotation view of NiV N, M and P proteins.** Hydrophobic amino acids are red and non-hydrophobic amino acids are displayed in blue. Movies were generated with Pymol software.
